# Leptin and adiponectin regulate the activity of nuclei involved in sleep-wake cycle in male rats

**DOI:** 10.3389/fnins.2022.907508

**Published:** 2022-07-22

**Authors:** Oscar Daniel Ramírez-Plascencia, Nadia Saderi, Skarleth Cárdenas-Romero, Fabio García-García, Carolina Peña-Escudero, Omar Flores-Sandoval, Lucia Azuara-Álvarez, Adrián Báez-Ruiz, Roberto Salgado-Delgado

**Affiliations:** ^1^Departamento de Fisiología Celular, Facultad de Ciencias, Universidad Autónoma de San Luis Potosí, San Luis Potosí, Mexico; ^2^Department of Neurology, Beth Israel Deaconess Medical Center, Harvard Medical School, Boston, MA, United States; ^3^Departamento de Biomedicina, Instituto de Ciencias de la Salud, Universidad Veracruzana, Veracruz, Mexico

**Keywords:** VLPO, sleep-wake, metabolism, obesity, circadian misalignment, timing of food intake, hypothalamus

## Abstract

Epidemiological and experimental evidence recognize a relationship between sleep-wake cycles and adiposity levels, but the mechanisms that link both are not entirely understood. Adipose tissue secretes adiponectin and leptin hormones, mainly involved as indicators of adiposity levels and recently associated to sleep. To understand how two of the main adipose tissue hormones could influence sleep-wake regulation, we evaluated in male rats, the effect of direct administration of adiponectin or leptin in the ventrolateral preoptic nuclei (VLPO), a major area for sleep promotion. The presence of adiponectin (AdipoR1 and AdipoR2) and leptin receptors in VLPO were confirmed by immunohistochemistry. Adiponectin administration increased wakefulness during the rest phase, reduced delta power, and activated wake-promoting neurons, such as the locus coeruleus (LC), tuberomammillary nucleus (TMN) and hypocretin/orexin neurons (OX) within the lateral hypothalamus (LH) and perifornical area (PeF). Conversely, leptin promoted REM and NREM sleep, including increase of delta power during NREM sleep, and induced c-Fos expression in VLPO and melanin concentrating hormone expressing neurons (MCH). In addition, a reduction in wake-promoting neurons activity was found in the TMN, lateral hypothalamus (LH) and perifornical area (PeF), including in the OX neurons. Moreover, leptin administration reduced tyrosine hydroxylase (TH) immunoreactivity in the LC. Our data suggest that adiponectin and leptin act as hormonal mediators between the status of body energy and the regulation of the sleep-wake cycle.

## Introduction

Sleep-wake disturbances play a major role in the deterioration of both physical and physiological health. Sleep disorders are frequently associated with several clinical conditions, including obesity (Ogilvie and Patel, 2017). Simultaneously, obesity impairs sleep architecture, sleep quality, and other sleep parameters both in humans (Resta et al., 2003; Bixler et al., 2005; Dagan et al., 2006; Li et al., 2017) and rodents (Jenkins et al., 2006; Mavanji et al., 2012; He et al., 2015; Luppi et al., 2017). In particular, obesity is associated with sleep disorders, such as obstructive sleep apnea (Bixler et al., 2005), decreased sleep quality, daytime sleepiness, and the development of poor sleep habits (Resta et al., 2003; Bixler et al., 2005).

The adipose tissue, far from being just an energy depot, is a highly active endocrine organ that takes part in body homeostasis secreting several peptide hormones, collectively known as adipokines (Fasshauer and Blüher, 2015). Adiponectin and leptin are two examples of adipokines that transmit information about the level of energy stored as fat tissue to the rest of the body, including the brain. In addition, they play a role in the regulation of food intake and energy expenditure. Notably, adiponectin plasma concentration is inversely correlated with fat mass. Adiponectin enhances the efficiency in the use of metabolic resources, like increasing glucose tolerance, insulin sensitivity, while also reduces energetic expenditure (Lee and Jianhua, 2014; Stern et al., 2016). Conversely, leptin communicates a positive energy balance to the brain, resulting in satiety and increased energy expenditure (Hyeong-Kyu and Ahima, 2015). The expansion of the adipose tissue in obesity is positively correlated to leptin synthesis and secretion. However, obese subjects develop an increase resistance against the anorexigenic and insulin-sensitizing properties of leptin (Fasshauer and Blüher, 2015).

Obesity impairment of sleep duration/quality has been associated with serum levels of adiponectin and leptin, but controversial results have been reported. For instance, some studies failed to find an association between adiponectin and sleep duration (Taheri et al., 2004; Robertson et al., 2013) while others found a positive correlation (Kotani et al., 2007; Sawamoto et al., 2016). Furthermore, an acute sleep restriction increased, reduced, or did not alter plasma adiponectin levels depending on sex and ethnicity (Simpson et al., 2010), and high adiponectin concentration has been related to chronic sleep restriction in diabetic patients (McHill et al., 2018; Oliveira et al., 2018). Similarly, there are no concordant results in the association of leptin serum levels and sleep parameters: no meaningful associations (Knutson et al., 2011; Capers et al., 2015; Soltanieh et al., 2021), negative correlations between leptin and sleep duration (Koo et al., 2013; Olson et al., 2016), or positive correlations with sleep time and quality have been reported (Pan and Kastin, 2014; Hirota et al., 2018). However, leptin receptor knockout rodents show changes in the circadian pattern of locomotor activity and sleep architecture, suggesting leptin signaling is involved in sleep regulation (Hsuchou et al., 2013; He et al., 2015). In addition, leptin receptors (LepR) are expressed in nuclei related to sleep induction in the anterior hypothalamus, such as the preoptic area (Zhang et al., 2011; Yu et al., 2016). Adiponectin and leptin plasma levels change during the day according to the energy requirements. Adiponectin concentration increases before the active phase onset (Gavrila et al., 2003; Scheer et al., 2010; Barnea et al., 2015), whereas leptin levels increase gradually during the active phase, peaking before the onset of the rest phase (Gavrila et al., 2003; Scheer et al., 2010). This evidence suggests that adiponectin might participate in promoting wakefulness during the active period, while leptin might gradually increase sleep pressure as its levels rise until the beginning of the sleep period, but little is now about the mechanism how adiponectin or leptin could act to regulate it. One possibility is that both adipokines could directly regulate sleep/wake promoting neurons, as those located in the ventro-lateral portion of hypothalamic preoptic area (VLPO; Szymusiak and McGinty, 2008; Pierre-Hervé, 2010), but this question remained unexplored.

To investigate this hypothesis, we examined the effects on wakefulness and sleep of local single administration of adiponectin or leptin in the sleep-promoting neurons located within the preoptic area including the VLPO. Since adiponectin and leptin exert opposite effects in metabolism, we expected opposite effects also in sleep regulation, then we consider for the injections time their circadian lowest blood levels and the major/lower sleep pressure trying to observe their major effect. Thus, adiponectin was injected at ZT1 and Leptin at ZT13 1 h after the lights turn on/off, respectively. The VLPO neurons are reciprocally interconnected with nuclei of the sleep-wake regulating circuitry, such as the lateral hypothalamus (LH), perifornical area (PeF), median preoptic nucleus (MnPO), suprachiasmatic nucleus (SCN), tuberomammillary nucleus (TMN), and locus coeruleus (LC) (Steininger et al., 2001; Chou et al., 2002; Lu et al., 2002; Sakurai et al., 2005; Pierre-Hervé, 2010; Saito et al., 2018). We also looked for immunoreactivity of the AdipoR1 and AdipoR2 in the VLPO, and also in the MnPO, SCN, TMN, and LC, and confirmation of the LepR immunoreactivity in the VLPO.

## Materials and methods

### Animals

Male Wistar rats (200–230 g of body weight) were individually housed in acrylic cages under a 12:12-h light-dark (LD) cycle. The onset of the illuminated period (lights-on) was defined as the Zeitgeber Time zero (ZT0). Controlled temperature (23°C ± 1) and free access to food (Laboratory Rodent Chow 5001, LabDiet, United States) and water were maintained during all the experiments.

The Ethical Committee approved all animal care and experimental procedures (CEID2014030), agreeing with the Mexican legislation for animal handling (NOM-062-ZOO-1999).

### Experiment 1: Immunohistochemistry to adiponectin and leptin receptors

To assess whether AdipoR1 and AdipoR2 are expressed in nuclei related to the sleep-wake cycle, a total of 8 rats were used. Half of them were sacrificed at ZT6 (*n* = 4) and the second half at ZT18 (*n* = 4). The brains were collected and paraffin-embedded for histological analysis (see “Tissue Processing and Immunohistochemistry” Section). Leptin receptor (LepR) were confirmed in three separated animals sacrificed at ZT6. The brains were collected and post-fixed for 24 h in 4% paraformaldehyde, then stored in 30% sucrose, 0.04% NaN_3_ (Amresco LLC, Solon, OH, United States) phosphate buffer saline solution (PBS 0.1 M, pH 7.4).

### Experiment 2: Modulation of the sleep and wake states by adiponectin and leptin

To record the sleep-wake states, a total of 20 rats were implanted with electroencephalogram (EEG) and electromyogram (EMG) electrodes. In addition, a 26-gauge stainless guide cannula (Plastic One, Roanoke, VA, United States) was unilaterally placed above the VLPO nucleus. During the implantation of electrodes and guide cannula, rats were deeply anesthetized with ketamine (13 mg/kg, PiSA, Hidalgo, Mexico) and xylazine (87 mg/kg, PiSA, Hidalgo, Mexico), and placed in a stereotaxic apparatus (Stoelting Co., Wood Dale, IL, United States). Four micro-screw electrodes were first implanted over the frontal and parietal bones to record EEG. Teflon-coated wires were placed bilaterally into both trapezius muscles to record EMG activity. Then, a guide cannula was placed into the VLPO (coordinates from the bregma: anterior = −0.3 mm, lateral = +1.3 mm and ventral = −8.5 mm) (Prerau et al., 2017). The EEG and EMG electrodes were connected to leads and plugged into a lightweight pedestal (pre-amplifier from Pinnacle Technologies, KS, United States). Guide cannula and electrodes were fixed onto the skull with dental cement (MDC Dental, Jal, Mexico). In addition, a sterilized temperature sensor (iButton Sensor-Temperature Logger; Maxim Integrated Products, San Jose, CA, United States) was inserted into the abdominal cavity. To reduce pain, inflammation and prevent infection, all rats were injected intramuscularly at the end of the surgery with ketorolac and enrofloxacin (3.5 and 0.2 mg/kg, respectively). After 10 days of recovery, rats were connected to a sleep recording system (Sirenia Acquisition System, Pinnacle Technologies, Lawrence, KS, United States) for 2 days of acclimatization using a flexible recording cable, which directly coupled the pre-amplifier to the electrical swivel (8409 Rat Commutator, Pinnacle Technologies). Core body temperature data were collected every 20 min, starting 3 days before the first adiponectin, leptin, or vehicle (0.9% saline solution). After euthanasia, temperature sensors were removed, and data were analyzed using the OneWireViewer software (version 3.15.49 2001–2010, Maxim Integrated Products, San Jose, CA, United States).

**FIGURE 1:**
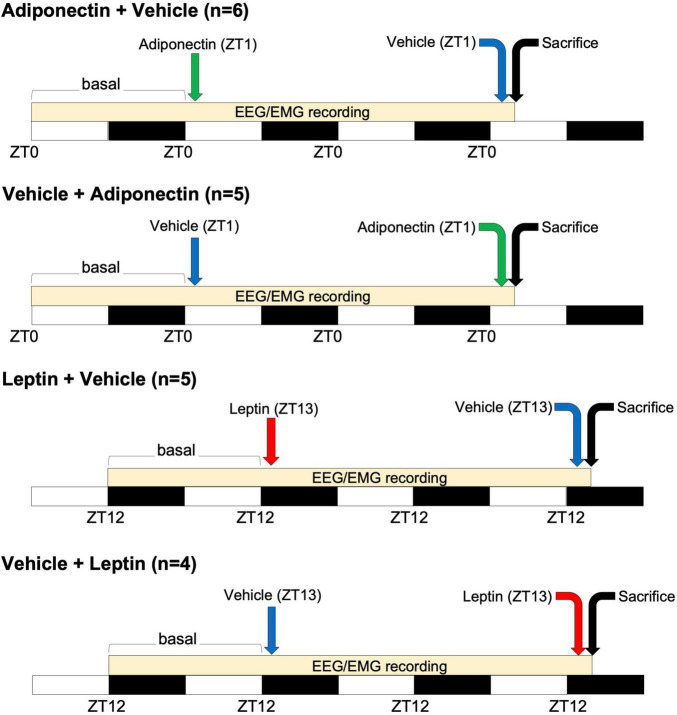
Time-course of adipokines administration. Four groups of animals received microinjection of adiponectin (at ZT1, green arrow) or leptin (at ZT13, red arrow) alternating with the administration of vehicle (before or after adipokines injection, blue arrow). White and black bars indicate the light and dark period, respectively. A difference of 48 h occurs between adipokine and vehicle administration. EEG/EMG recording occurs at the onset of that basal period, during the following 23 h after the first microinjection and along the 2 h after the second one. Animals were sacrificed two h after the second microinjection as indicated by the black arrow. ZT, Zeitgeber time; EEG, electroencephalogram; EMG, electromyogram.

Before starting experimental procedures, rats were randomly assigned to one of the following protocols: Adiponectin + Vehicle (*n* = 6), Vehicle + Adiponectin (*n* = 5), Leptin + Vehicle (*n* = 5), and Vehicle + Leptin (*n* = 4). Following baseline sleep and temperature recording, rats assigned to Adiponectin + Vehicle or Vehicle + Adiponectin were briefly immobilized to remove the dummy stylets and insert the injection cannula. Once rats were allowed to move freely again, 1 μL of either adiponectin (1 μM, globular form, Catalog 003-17, Phoenix Pharmaceuticals, CA, United States) or vehicle were infused by a perfusion pump (Harvard Apparatus, model pump elite 11) at a flow rate of 200 nL/min. Injections started at ZT1 when the levels of adiponectin are low and the sleep pressure high. The adiponectin concentration used was previously tested in similar protocols and determined according (Hoyda et al., 2009; Klein et al., 2011). To allow diffusion and avoid reflux, the injection cannula was left in place for 5 min post-injection. Subsequently, the animals were once more briefly immobilized to remove the injection cannula and reinsert the dummy stylets. After the first microinjection, sleep and wakes states were recorded for the next 23 h. After a day of clearance, rats were switched to receive their counterbalance protocol administration (vehicle or adiponectin), followed by 2-h of sleep-wake states and temperature recording. At the end of the protocol, rats were euthanized for histological analysis (see Figure 1 for timing details). Rats assigned to receive microinjections of Leptin + Vehicle or Vehicle + Leptin (5 μM, Leptin recombinant, Catalog 003-17, Phoenix Pharmaceuticals, CA, United States) underwent an identical experimental protocol as the one described for Adiponectin, except the injection took place at ZT13 when blood levels of leptin and the sleep pressure were low (1-h after lights-out) (Figure 1). Leptin concentration was determined according to previous reports using similar protocols (Watanobe, 2002; Bruijnzeel et al., 2011; Verhagen et al., 2011).

Food intake was measured in three occasions: during baseline, after the first injection (for 24 h each), and lastly, after the second counterbalance injection (for 2 h).

The behavioral states of wakefulness, NREM, or REM sleep were manually scored offline in 10 s epochs using the Sirenia Sleep Analysis software (SSA, Pinnacle Technologies, Lawrence, KS, United States). Wakefulness was identified by desynchronized EEG activity coupled with phasic/high EMG activity. NREM sleep scoring corresponded to epochs featuring high amplitude (100–200 μV) and slow frequency (0.5–4.0 Hz) EEG waves, as well as low muscle tone. REM sleep was identified by theta (4–8 Hz) EEG activity and loss of muscle tone. Sleep latency was calculated from the time of injection until the first consolidated NREM or REM sleep (at least 30 s/3 consecutive epochs). Power density of the EEG signal (μV^2^) during NREM sleep was calculated using fast-Fourier transformation analyzed in 0.25 Hz bins in the frequency range of 0.25–20.0 Hz with Sleep Sign Software (3.0, Kissei Comtec Co., Nagano, Japan). The average power of EEG in NREM sleep epochs was calculated during the 2 h after the injections and at baseline at the same temporal points.

### Brain tissue collection

Rats were euthanized by an overdose of sodium pentobarbital (65 mg/ml; Sedalpharma, Pet’s Pharma, Mexico), then perfused with 0.9% saline solution followed by 4% paraformaldehyde (Sigma-Aldrich Corp., St. Louis, MO, United States) diluted in phosphate buffer (PB 0.1 M, pH 7.4). Rats assigned to the Adiponectin + Vehicle or Vehicle + Adiponectin were euthanized at ZT3, while animals in protocols Leptin + Vehicle or Vehicle + Leptin were euthanized at ZT15 on the last day of the recordings (see Figure 1). Brains were collected and post-fixed for 24 h in 4% paraformaldehyde, and then stored in 30% sucrose, 0.04% NaN_3_ (Amresco LLC, Solon, OH, United States) phosphate buffer saline solution (PBS 0.1 M, pH 7.4) until further tissue processing.

### Tissue processing and immunohistochemistry

Paraffin-embedded brain tissue from experiment 1 was sectioned into 16 μm-thick coronal slices (HM 325 Rotary Microtome - Thermo Fisher Scientific, Walldorf, BW, DE), which were placed over with poli-L-lisine-covered slides (Sigma-Aldrich Corp.) for paraffine-immunohistochemistry (IHC-p). Two sets from each brain were incubated with a goat anti-AdipoR1 primary antibody (1:200; sc-46748, Santa Cruz Biotechnology, Dallas, TX, United States) or goat anti-AdipoR2 primary antibody (1:200; ab77613, Abcam, Cambridge, United Kingdom) for 48 h. Previously, the antibodies were diluted in Tris Buffered Saline (TBS, Sigma-Aldrich Corp.) with 0.1% TWEEN 20 (Sigma-Aldrich Corp.), and their specificity were evaluated by western blot in hypothalamic tissue and no primary antibody as negative control for the brain IHC staining (Supplementary Figure 1). After three times washing with TBS-Tween, sections were incubated with a biotinylated donkey anti-goat antibody (1:200; 705-065-147, Jackson ImmunoResearch, West Grove, PO, United States) for 2 h, and subsequently incubated with the Avidin-Biotin complex solution (1:500; Vector laboratories, Burlingame, CA, United States) for an additional 2 h. The final reaction was visualized with a TBS solution containing 0.025% diaminobenzidine (Sigma-Aldrich Corp.) and 0.01% hydrogen peroxide (J. T. Baker, Mexico).

**FIGURE 2:**
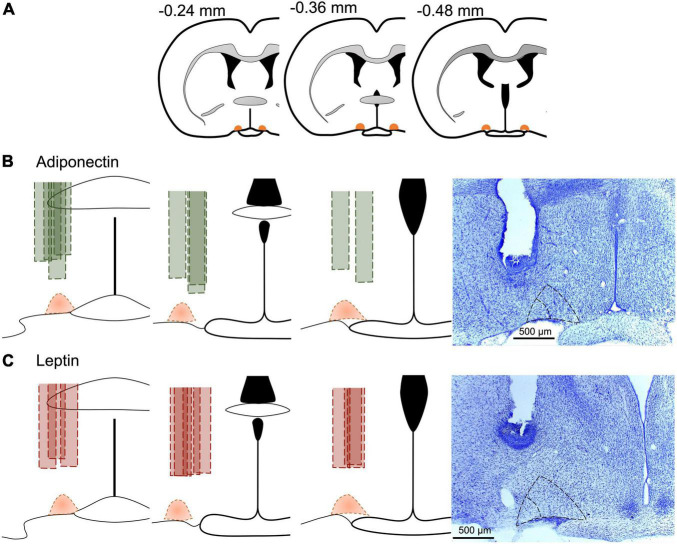
Cannula position in experimental animals. **(A)** Schematic representation of the anteroposterior reference of VLPO, and the site of the cannula in each animal included for **(B)** adiponectin in green or **(C)** leptin in red. Dotted area in orange represents the VLPO extension as described in the brain atlas (Paxinos and Watson, 2007). Representative images at the right for the site of the cannula for each adipokine, core VLPO is represented within the inner dotted triangle, while the bigger dotted triangle include the extended VLPO. Brains were stained with cresyl violet for better visualization.

Brains for Leptin receptor (LepR) confirmation and from experiment 2 were cut at −20°C into 40 μm-thick coronal sections (Microtome Cryostat Microm HM 525 – 387779 – Thermo Fisher Scientific) and collected in four alternated series. In the animals from experiment 2, the position of the cannula within the VLPO was validated by staining a representative sample of slices for 5 min in 0.02% cresyl violet solution (Figure 2).

Antibodies used for LepR and in experiment 2 were diluted in TBS with 0.25% gelatin (Merck KGaA, Darmstadt, Germany) and 0.5% Triton X-100 (Sigma-Aldrich Corp.). The LepR antibody specificity was tested by western blot in kidney tissue (Supplementary Figure 3C). LepR was confirmed using the antibody Rabbit anti-LepR polyclonal (1:2000; ab5593 Abcam, Cambridge, United Kingdom), incubating the brains in free-floating for 24 h. The brains were rinsed three times in PBS and incubated for 2 h with a biotinylated donkey anti-rabbit antibody (1:200; Jackson ImmunoResearch), followed by the Avidin-Biotin complex also incubated for 2 h. The final reaction was visualized as indicated previously.

In the brains from experiment 2, two sets of free-floating sections were first incubated with a rabbit anti-c-Fos primary antibody for 24 h (1:2500; Santa Cruz Biotechnology, Dallas, TX, United States), and then followed the same protocol described for LepR, but 0.1% NiNH_4_SO_4_ was added to the DAB-peroxide solution used for visualization (Sigma-Aldrich Corp.). After IHC examination of c-Fos in brains from experiment 2, the slices including the LH and PeF were selected and incubated overnight with either goat anti-Orexin A primary antibody (1:4000; Santa Cruz Biotechnology) from the first set of brains, or with goat anti-MCH primary antibody (1:2000; Santa Cruz Biotechnology) from the second set of slides. The sections were incubated with donkey anti-goat second antibody (1:200) the day after, followed by the Avidin-Biotin complex and the DAB-peroxide solution. Additionally, a third set of sections was incubated with rabbit anti-Tyrosine Hydroxylase (TH) antibody (1: 4000; ab112 Abcam, Cambridge, United Kingdom), and a fourth set was incubated with mouse anti-GAD65 antibody (1: 2000; ab26113 Abcam, Cambridge, United Kingdom). In both cases, the following steps were the same as previously described using but the secondary antibody used for GAD65 was donkey anti-mouse (1:200; 715-065-150 Jackson ImmunoResearch, West grove, PO, United States).

Sections were mounted in gelatinized slides, dehydrated with a growing concentration of ethanol, cleared with xylene (Golden Bell, Zapopan, JAL, Mexico), and cover slipped with Entellan (Merck KGaA, Darmstadt, Germany) for visualization under the light microscope.

### Cell counting

Anterior, medial and posterior plane levels (one section per level) were selected for each nucleus. The area were delimited and immunoreactive cells tallied according with the Paxinos and Watson rat brain atlas for VLPO (−0.24, −0.36, and 0.48), for TMN (−3.84, −4.08, and −4.32), for LC (−9.68, −9.80, and −10.04), LH/PeF (−1.88, −2.56, and −3.30), and MnPO (0.36, 0.12, and −0.12) (the numbers correspond to the anterior/posterior level to the bregma, as is indicated in the stereotactic atlas, Paxinos and Watson, 2007). Sections were visualized and captured bilaterally under a light microscope Leica DM500, equipped with a camera (Leica ICC50 HD) and the Leica Application Suite software version 3.0 (Leica Microsystem Limited, Switzerland, 2013). After selecting the area and subtracting background, immunoreactive cells for c-Fos were analyzed using the following settings: threshold (0, 195), particle size (500–1,800), and circularity (0.0–1.0) parameters using the image J imaging analysis software (1.47v, National Institutes of Health, United States). The average area (in pixels) was standardized in each nucleus for using it as reference in quantifications. GAD65 and TH immunoreactivity was quantified as integrated optical density (IOD) using image J, following the developer recommendation, evaluated in gray scale, and expressed as arbitrary units (AU). c-Fos/OX and c-Fos/MCH co-labeling were examined under a light microscope (Carl Zeiss Axio Lab A1 and the ZEN blue edition program, 2011) and manually counted the single- and co-labeling using the multipoint tool in Image J program, considering the total of OX or MCH neurons (single- plus double-labeled cells) in every nucleus as 100%. Random images were blind counted with the same protocol by a second person to evaluate the intrasubject variability (ICC = 0.884, 95% CI: 0.725–0.953, *n* = 20).

### Statistical analysis

Data are represented as mean ± standard error of the mean (SEM). All data sets fulfilled the parametric criteria for Brown–Forsythe (homo/heteroscedasticity) and Shapiro–Wilk (fit to normal distribution) as well as these same criteria for the residuals of the models. Food intake, mean body temperature, and total sleep parameters were analyzed with one-way ANOVA, followed by a *post hoc* multiple comparisons Tukey test. Continuous recording of body temperature and sleep/wake data, and power density from EEG data were analyzed with two-way ANOVA with a factor for group and a factor for time (no assumption of sphericity, Geisser–Greenhouse correction used instead), followed by a *post hoc* multiple comparisons Tukey test. Cell counting and optical density analysis were analyzed with a Student *t*-test. The 2-h data analysis includes the first 2 h of 24-h recording and the 2-h recordings at the end of protocol. In all cases, significance values α were set at 0.05. Statistical analyses were performed with the GraphPad Prism software version 8.

## Results

### AdipoR1, AdipoR2, and LepR immunoreactivity in ventrolateral preoptic nuclei and other sleep-wake regulatory nuclei

**FIGURE 3:**
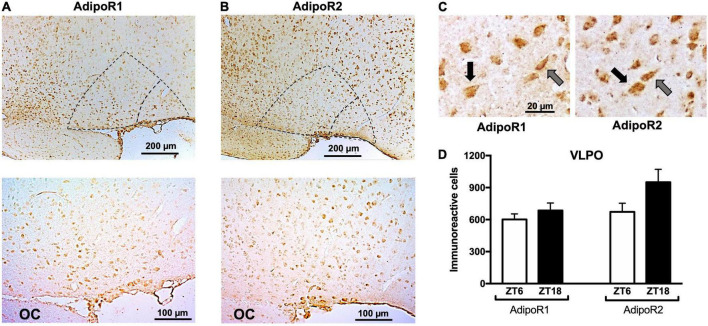
Immunoreactivity of adiponectin receptors in the VLPO. Representative immunoreactivity of **(A)** AdipoR1 or **(B)** AdipoR2 at 10× (upper) and 20× magnification (lower). In both cases, core VLPO is observed in the inner dotted triangle, and the bigger triangle represents the extended VLPO. **(C)** Pyramidal (black arrows) and fusiform (gray arrows) morphology were observed in AdipoR1 and AdipoR2. **(D)** AdipoR1 or AdipoR2 immunoreactivity positive cells at ZT6 or ZT18. VLPO, ventrolateral preoptic nucleus; OC, optic chiasm.

Immunohistochemistry shows LepR, AdipoR1, and AdipoR2 immunoreactivity is expressed in the VLPO and in several other areas of the brain (Figure 3 and Supplementary Figures 1–3). VLPO cells expressing AdipoR1 and AdipoR2 display either a fusiform or pyramidal shape, similar to the morphology of interneurons and projecting neurons, respectively (Figure 3C). No change in AdipoR1 and AdipoR2 immunoreactivity in the VLPO was observed between ZT6 and ZT18 (Figure 3D). Within the hypothalamus, the expression of both receptors was detected especially in arcuate nucleus (ARC), paraventricular nucleus (PVN), SCN, TMN, and MnPO (Supplementary Figures 1–3). Out of the hypothalamus, locus coeruleus nucleus (LC) expresses both adiponectin receptors (Supplementary Figure 2).

### Food intake and core body temperature changes after adiponectin or leptin administration

**FIGURE 4:**
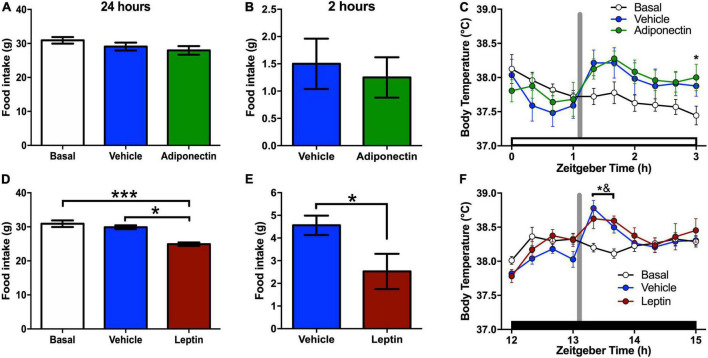
Food intake and temperature responses after adipokines administration. **(A,D)** Food intake after 24 h of adipokines or vehicle administration compared with baseline data. **(B,E)** Food intake after 2 h of adipokines or vehicle administration prior to euthanasia. **(C,F)** Body core temperature after **(C)** adiponectin or **(F)** leptin administration. In food intake graphs: *P < 0.05, ***P < 0.001. In body temperature graphs: white bar, light phase; dark bar, dark phase; vertical gray line, a moment of the adipokine or vehicle administration; *P < 0.05 Adipokine vs. basal; ^&^*P* < 0.05 vehicle vs. basal.

With respect to the vehicle or baseline, no changes in food intake were observed 2 or 24 h after adiponectin infusions [24 h: *F*_(2,24)_ = 1.562, *p* = 0.23; 2 h: *T* = 0.405, *p* = 0.697; Figures 4A,B]. In contrast, leptin administration induced a significant decrease in food intake detectable 2 h after the injection and which persisted for 24 h [24 h: *F*_(2,24)_ = 6.758, *p* = 0.004; 2 h: *T* = 2.427, *p* = 0.045; Figures 4D,E]. Noteworthy adiponectin was given at light-on onset, while leptin was administered at the beginning of the lights-out period. Laboratory rats consume most of the food during the dark period.

All groups presented a clear circadian rhythm in core body temperature, with higher values during the dark phase (Supplementary Figure 4). The administration of adiponectin or vehicle leads to an upsurge in core temperature in comparison with basal records which did not differ significantly between treatments (Figure 4C). Two-ways ANOVA for 2h recordings indicated a significant effect of time [*F*_(9,216)_ = 4.385, *p* < 0.001], and time*protocol interaction [*F*_(18, 216)_ = 2.722, *p* < 0.001], but not for protocol [*F*_(2,24)_ = 1.203, *p* = 0.317], Similarly, both leptin and vehicle administration elevated core body temperature during the first hour after injection compared to baseline (Figure 4F). Two-way ANOVA for 24 h recording indicate an effect in time [*F*_(72,1080)_ = 30.44, *p* < 0.001] and time*protocol interaction [*F*_(144,1080)_ = 1.397, *p* = 0.002], but not for protocol alone [*F*_(2,15)_ = 0.6175, *P* = 0.552].

### Adiponectin administration in ventrolateral preoptic nuclei reduced REM sleep and increased wakefulness

**FIGURE 5:**
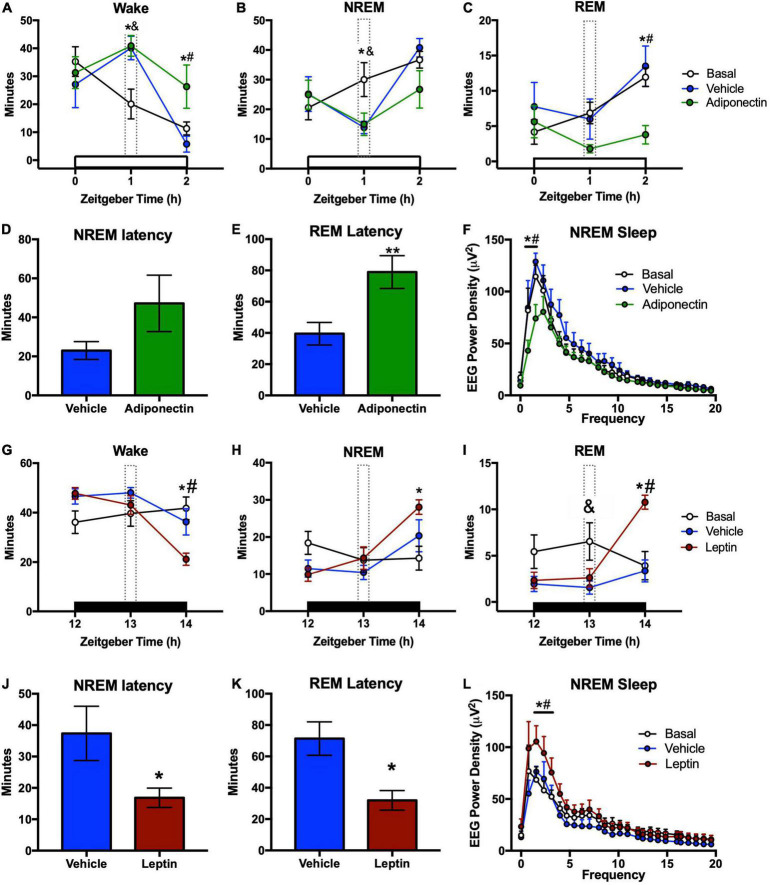
Sleep-wake times and latency after adipokines administration. Wake, NREM, and REM time after **(A–C)** adiponectin or **(G–I)** leptin administration. **(D)** NREM and **(E)** REM latencies from the moment of **(D,E)** adiponectin or **(J,K)** leptin microinjection. Change in delta power during NREM sleep after **(F)** adiponectin or **(L)** leptin administration. In sleep-wake graphs: white bar, light phase; dark bar, dark phase; dotted line, hour of adipokine administration; *P < 0.05 adipokine vs. basal; ^&^*P* < 0.05 vehicle vs. basal; ^#^*P* < 0.05 adipokine vs. vehicle. In latency graphs: *P < 0.05, **P < 0.01.

Circadian distribution of sleep-wake states was observed during baseline (Supplementary Figure 5). Adiponectin increased wake and reduced REM sleep during the second hour after the administration, but the 24 h distribution of wake and sleep did not change (Figures 5A–C and Supplementary Figure 5). Two-way ANOVA for wake time indicate a significant effect in Time (*F*_2,42_ = 12.1, *p* < 0.001) and time*Protocol interaction (*F*_4,42_ = 3.253, *p* = 0.0206), but no for Protocol factors (*F*_2,21_ = 2.837, *p* = 0.0812); NREM sleep was significant for time factor (NREM: *F*_2,42_ = 9.959, *p* < 0.001) and time*protocol interaction (NREM: *F*_4,42_ = 2.85, *p* = 0.0354), but not protocol (NREM: *F*_2,21_ = 1.339, *p* = 0.2837); REM sleep is significant for time (*F*_2,42_ = 5.751, *p* < 0.001) and protocol (REM: *F*_2,21_ = 5.043, *p* = 0.2624) but not for time*protocol interaction (*F*_4,42_ = 2.399, *p* = 0.0651).

Moreover, adiponectin delayed REM sleep onset when compared with vehicle (*t* = 1.201, *p* = 0.249), but did not change NREM sleep latency (*t* = 2.718, *p* = 0.016; Figures 5D,E). Adiponectin reduced the average EEG power density during NREM sleep in the 2 h after administration, in comparison with vehicle injection and the basal line (Figure 5F). The two-way ANOVA indicates a significant effect of Time (*F*_25,364_ = 46.66, *p* < 0.001) and Protocol (*F*_2,364_ = 16.68, *p* < 0.001), but no effect of Time*Protocol interaction (*F*_50,364_ = 1.207, *p* = 0.17).

### Leptin administration in the ventrolateral preoptic nuclei promoted sleep

In contrast to the alerting effects of adiponectin, leptin injection in the VLPO significantly decreased the wake time. As compared to vehicle, both NREM and REM sleep were significantly increased during the second hour after administration [Wake: *F*_(2,40)_ = 10.98, *p* < 0.001; NREM: *F*_(2,40)_ = 11.24, *p* < 0.001; REM: *F*_(2,40)_ = 5.717, *p* = 0.006, Figures 5G–I]. Leptin did not increase sleep amounts during the first hour or throughout the next 24 h (Figures 5G–I and Supplementary Figure 5). Latencies to NREMS and REM sleep were also significantly delayed (NREM sleep, *t* = 2.507, *p* = 0.026; REM sleep, *t* = 3.389, *p* = 0.004; Figures 5J,K). Sleep promoting effects of leptin were confirmed by the increased average EEG power density measured during NREMS during the 2 h after administration (Figure 5I). Two-way ANOVA indicates a significant effect on Time (*F*_25,416_ = 20.14, *p* < 0.001) and Protocol (*F*_2,416_ = 12.15, *p* < 0.001), but no effect of Time*Protocol interaction (*F*_50,416_ = 0.703, *p* = 0.936).

### c-Fos immunoreactivity levels in wake-sleep regulatory nuclei after leptin or adiponectin administration

**FIGURE 6:**
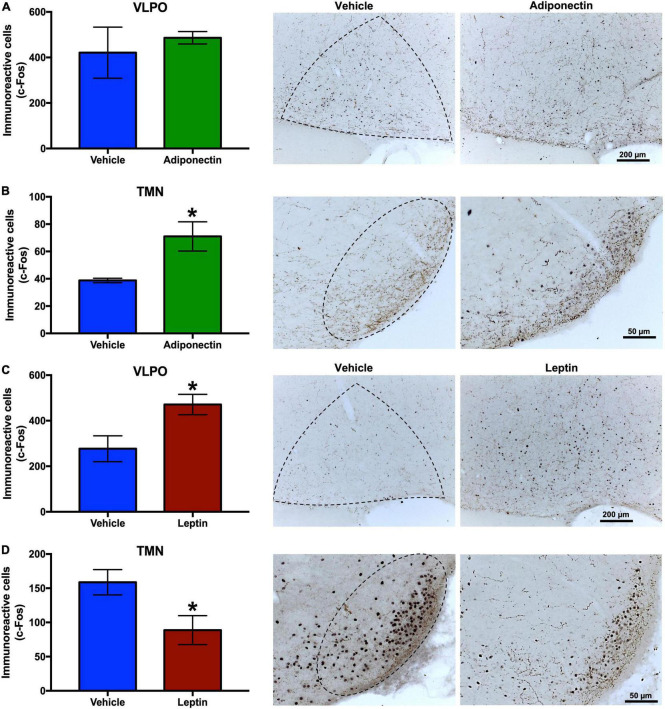
c-Fos immunoreactivity in VLPO and TMN after adipokines or vehicle administration. Immunoreactive cells to c-Fos in VLPO and TMN 2 h after **(A,B)** adiponectin or **(C,D)** leptin administration, and representative images (right columns, dashed lines border the nuclei area). Adipokine vs. vehicle *P < 0.05. VLPO, ventrolateral preoptic nucleus; TMN, tuberomammillary nucleus.

**FIGURE 7:**
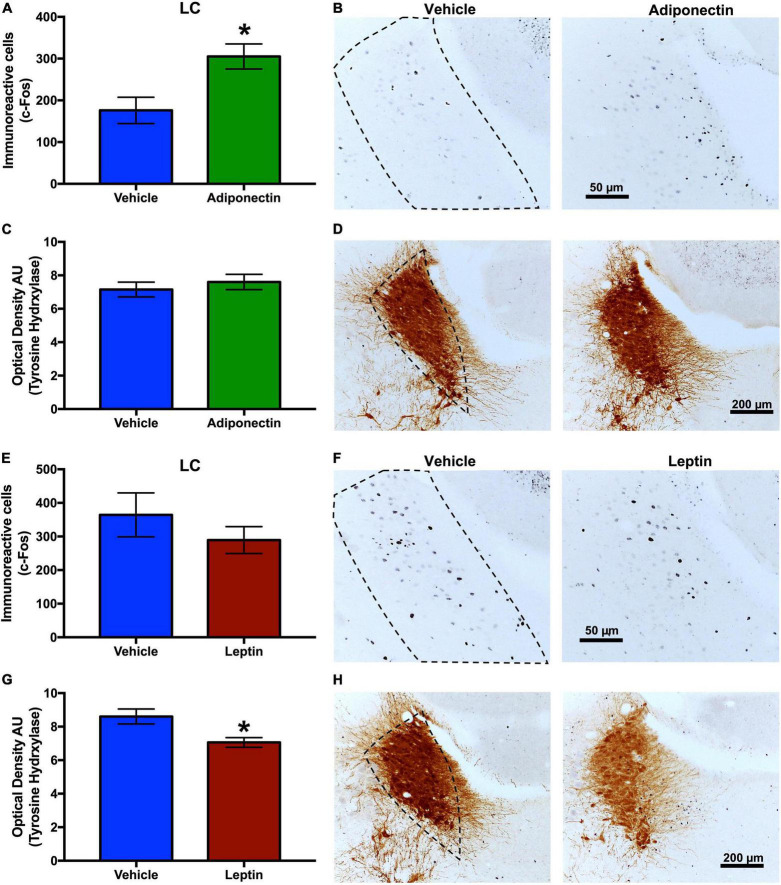
c-Fos and TH immunoreactivity in the LC after adipokines or vehicle administration. Immunoreactive cells to c-Fos in LC 2 h after **(A,B)** adiponectin or **(E,F)** leptin administration. Immunoreactive cells to TH in LC 2 h after **(C,D)** adiponectin or **(G,H)** leptin administration. Representative images (right columns, dashed lines border the nucleus area). Adipokine vs. vehicle *P < 0.05. LC, locus coeruleus.

The neuronal response, evaluated by c-Fos immunoreactivity, were tallied in the VLPO, TMN, and LC nuclei 2 h after the administration of adiponectin, leptin, or vehicle. Adiponectin significantly increased the activity of TMN, although no changes were observed in the VLPO (Figures 6A,B). Leptin induced a significant increase in c-Fos immunoreactivity in VLPO (Figure 6C) while reduced the activity of neurons located within wake-promoting nuclei as TMN (Figure 6D). In the LC, adiponectin administration increased c-Fos immunoreactivity (Figures 7A,B), while the injection of leptin did not affect the number of c-Fos labeled neurons (Figures 7E,F).

### Opposite effects of adiponectin and leptin on activity of the orexin neurons and melanin concentrating hormone neurons

**FIGURE 8:**
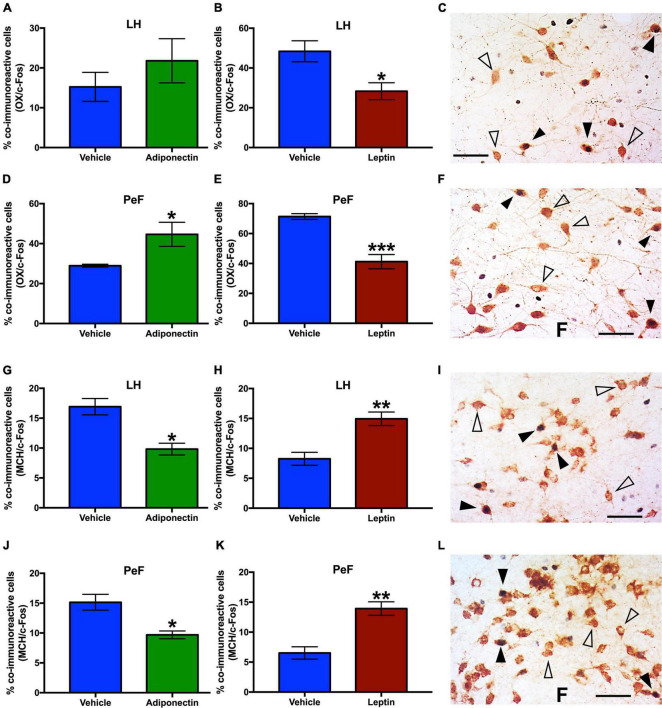
OX-c-Fos and MCH-c-Fos co-immunoreactivity in LH and PeF nuclei after adipokines or vehicle administration. Percentage of OX/c-Fos co-expression in **(A–C)** LH and **(D–F)** PeF area 2 h after adiponectin or leptin administration, respectively. Percentage of MCH/c-Fos co-expression in **(G–I)** LH or **(J–L)** PeF area 2 h after adiponectin or leptin administration, respectively. Representative images from the double immunoreactivity are shown in the right panels **(C,F,I,L)**. Open triangles, OX or MCH neurons, black triangles, OX/c-Fos or MCH/c-Fos co-immunoreactivity, and black arrow c-Fos immunoreactive cells. Adipokines vs. vehicle *P < 0.05, **P < 0.01, ***P < 0.001. OX, Orexin; MCH, melanocortin; LH, lateral hypothalamus nucleus; PeF, perifornical area.

The total number of OX or MCH neurons was similar among rats given adiponectin or leptin in LH and PeF (Supplementary Figure 6). After adiponectin administration, the percent of OX/c-Fos co-labeled neurons significantly increased in the PeF region (Figure 8D), while the percent of MCH/c-Fos co-labeled cells decreased in this region in the LH (Figures 8G,J). Adiponectin injection did not change the number of OX/c-Fos positive neurons in the LH (Figure 8A). In contrast, after leptin administration, the percentage of OX/c-Fos co-labeled neurons was reduced in both LH and PeF (Figures 8B,E), meanwhile MCH/c-Fos co-labeled neurons were increased in both nuclei (Figures 8H,K).

### Effects of adiponectin and leptin on GAD65 and tyrosine hydroxylase immunoreactivity in the locus coeruleus and other brain regions

The effect of leptin or adiponectin administration on GAD65 and TH immunoreactivity was evaluated by optical density in the VLPO, MnPO, TMN, and LC nuclei (Supplementary Figure 7). Adiponectin administration did not change optical density of GAD65 immunoreactive signal, in any brain region studied with the exception of the LC where it significantly increased (Table 1 and Supplementary Figure 7D). The administration of leptin significantly increased the optical density of GAD65 in the MnPO (Table 2 and Supplementary Figure 7F). In the LC, adiponectin did not change tyrosine hydroxylase immunoreactivity (Figures 7C,D), while leptin significantly reduced it (Figures 7G,H).

**TABLE 1:** Optical density of GAD65 expression in adiponectin protocols.

Nucleus	Vehicle (mean ± *SEM*)	Adiponectin (mean ± *SEM*)	*p*
VLPO	3.413 ± 0.07	3.484 ± 0.08	0.5659
MnPO	1.752 ± 0.02	1.799 ± 0.02	0.1940
TMN	2.799 ± 0.08	2.795 ± 0.05	0.9654
LC	7.589 ± 0.14	8.168 ± 0.11	0.0143*

*Significance against the vehicle group.

**TABLE 2:** Optical density of GAD65 expression in leptin protocols.

Nucleus	Vehicle (mean ± *SEM*)	Leptin (mean ± *SEM*)	*p*
VLPO	3.505 ± 0.09	3.449 ± 0.06	0.6669
MnPO	1.226 ± 0.01	1.32 ± 0.02	0.0276*
TMN	3.081 ± 0.09	2.8 ± 0.07	0.0572
LC	6.754 ± 0.2	6.758 ± 0.28	0.9911

*Significance against the vehicle group.

## Discussion

Many studies have revealed the effects of metabolic alterations (such as obesity) over the sleep-wake cycle, but only a few explored the mechanism underlying this interplay. In the present study, we show that the receptors for the adipose tissue-derived hormones adiponectin (AdipoR1 and AdipoR2) and leptin (LepR) are present a prominent sleep-promoting nucleus, the VLPO. Furthermore, adiponectin and leptin administration into the VLPO changes the amount of sleep and wake during the second hours after administration. Notably, adiponectin induces wakefulness and inhibits REM sleep by reducing its total time and increasing its latency, while leptin increases REM and NREM sleep total time and reduces their latency. Moreover, the activity of other neurons reciprocally connected with the VLPO, such as in the LH, PeF, TMN, and LC, was also significantly changed by adiponectin or leptin microinjection in the preoptic area over the VLPO. Our data suggest that the adipose tissue influences sleep and wake regulation via the adiponectin and leptin signaling in the ventral lateral preoptic area. This hormonal communication might be involved in the sleep alterations observed in obesity.

Adiponectin receptors 1 and 2 exert functional differences including affinity, signaling pathways, and changes in the membrane phospholipid composition (Kadowaki et al., 2006; Yamauchi et al., 2014; Pilon, 2021). For this reason, we decided to explore whether they were differentially expressed in the preoptic area and other important sleep/wake regulating nuclei. Within the preoptic area, adiponectin receptors have been reported to be expressed in neuronal subsets involved in thermoregulation (Klein et al., 2011). Here, we report for the first time that AdipoR1 and AdipoR2 are expressed in the VLPO, in both the core and the extended areas of this nucleus (Figures 3A,B). We found AdipoR1 and AdipoR2 in fusiform and pyramidal shaped cells, which have been suggested to be interneurons and neurons projecting outside the VLPO, respectively (Figure 3C; Gallopin et al., 2000; Matsuo et al., 2003; Yu-Wei et al., 2010). Few authors reported that adiponectin receptors are co-expressed with glia markers (Guillod-Maximin et al., 2009; Shen et al., 2014), it might also occur in the VLPO, but this needs further confirmation. Since the receptors expression might have a circadian component, we explore the expression of them during the lights and dark period, but no differences were observed. Most investigations relating to adiponectin receptors have been focused on nuclei involved in energy homeostasis, such as the ARC and PVN nuclei, where AdiporR1 and AdipoR2 expression is the highest (Supplementary Figure 2; Coope et al., 2008; Guillod-Maximin et al., 2009). We observe that, in addition to the ARC and PVN, adiponectin receptors are also expressed in several areas involved in sleep-wake regulation and circadian rhythms, including the MnPO, SCN, TMN, and LC (Supplementary Figure 2).

Regarding the effects on feeding behavior, we found that adiponectin administration in the preoptic area within the VLPO did not promote food intake, although it increased wakefulness. Contradictory results have been reported about the feeding response after adiponectin administration in the brain. In some studies, food consumption has increased (Kubota et al., 2007), while in others decreased (Coope et al., 2008) or has remained without change (Miyatake et al., 2015). It has been suggested that the sensitivity of the hypothalamus to adiponectin might depend on energy availability, having an anorexigenic or orexigenic effect in presence of high or low glucose levels, respectively (Suyama et al., 2016). Our rats received adiponectin at the beginning of the light phase and have had free access to food during the previous 12 h, which might explain why adiponectin did not trigger food consumption. As an alternative, our results suggest adiponectin regulation of food intake depends on other hypothalamic nuclei outside the VLPO, such as the ARC.

Intravenous (IV) administrated adiponectin reduces UCP-1 expression and oxygen consumption in mice (Kubota et al., 2007), while intracerebroventricular (ICV) administration increases UCP-1 and energy expenditure in ob/ob mice (Qi et al., 2004), but failed to exert an effect in wild type rats (Messina et al., 2018). Similar to Klein et al. (2011), we found AdipoR1 and AdipoR2 expressed in the thermosensitive nucleus MnPO (Supplementary Figure 2), but no distinguishable response was observed between Adiponectin and vehicle microinjection (Figure 4), so the increase in body temperature in both groups was mostly related to the handling at the moment of the microinjections than the hormone action. KO mice models show that adiponectin plays a central role in UCP-1 expression and thermoregulation by direct activation of brown adipose tissue (Qiao et al., 2014; Wei et al., 2017), but the adiponectin regulation of temperature seem to occur only under specific circumstances (as in ob/ob mice) or is secondary to the stimulation of energy expenditure, probably associated to other hypothalamic areas and not to the ventral lateral preoptic region.

Adiponectin administration in the preoptic area within the VLPO induces an acute increase in wakefulness and reduces REM sleep time and latency. No changes were observed on the NREM time after adiponectin administration, but interestingly, the alerting effects of adiponectin is also observed in the reduction of the delta power (Figure 5). Delta power is one EEG parameter associated with the sleep quality/intensity, the reduction in delta power suggest that adiponectin might participate in the quality of sleep, but this hypothesis needs further confirmation. However, we cannot discard that the concentration of the protein injected, or the acute injection instead of chronic administration, is not enough to reduce significatively the NREM time and latency. Furthermore, the reduction of the delta power occurs at the beginning of the rest period. For instance, in patients with severe Obstructive Sleep Apnea (OSA), the adiponectin levels in serum are higher before sleep time than after (Nakagawa et al., 2008), and OSA subjects show fragmented sleep with overall low-quality (Mutairi et al., 2014; Lu et al., 2019) in agreement with the hypothesis that adiponectin affects the quality of sleep.

Since most of the previous reports focused on how sleep affect adiponectin levels, to date, no study has evaluated the influence of adiponectin on sleep or wake. Our results offer the first affirmative evidence that adiponectin is a wake-promoting hormone. Interestingly, alterations in the signaling of AdipoR1 in a mouse model of metabolic syndrome produces circadian alterations in locomotor activity (Hashinaga et al., 2013). Also, AdipoR1-KO mice display a reduction in locomotor activity, while AdipoR2-KO shows the opposite (Bjursell et al., 2007); thus, it is possible that the increase in wake time is mediated by AdipoR1.

LepR has been described in nuclei related to the homeostatic control, such as the ARC, PVN, and LH, among others within and outside of the hypothalamus. LepR are expressed in the preoptic area, with the densest expression in the MnPO, which has sleep-promoting cells, and is sparsely found within neurons in the lateral-ventral preoptic area (Zhang et al., 2011; Yu et al., 2016). Here we have confirmed the presence of lepR in the VLPO (Supplementary Figure 3). Microinjection of leptin in the lateral preoptic area including the VLPO significantly decreases food intake after 2 and 24 h. Our findings are in agreement with the key anorexic function already described for this hormone (Pandit et al., 2017; Zhang and Chua, 2018). In addition, our results are consistent with the hypothesis that the ventral lateral portion of the preoptic area might participate in the regulation of food intake by leptin.

The thermogenic effect of leptin is well established. Leptin administration increases body core temperature by activation of brown adipose tissue, while leptin-deficient mice ob/ob display a reduction in basal body temperature and in response to cold exposure, which can be normalized by exogenous leptin administration (Enriori et al., 2011; Rezai-Zadeh et al., 2014; Fischer et al., 2016). Our results also show an increase in the core body temperature although the hypothermic effect of leptin was no different from the vehicle. We believe that the lack of effect could be a consequence of animal manipulation during the microinjection. In addition, leptin microinjection was made during the active phase of rats, when the temperature was at its physiological ceiling, likely masking the effect of leptin. It has been also reported that the activation of neurons containing leptin receptors in the preoptic area reduces body temperature (Barnea et al., 2015) and food intake (Yu et al., 2018). Here, we did not observe the temperature effect of leptin, but our results are consistent with those regarding food intake.

We also found that leptin microinfusion in the preoptic area over the VLPO promoted NREM and REM sleep, by increasing their total time, reducing their latencies, and increasing NREM sleep delta power when compared to the vehicle injection and baseline recording (Figures 5G–L). Similar results showing an increase of NREM and delta power after leptin administration were observed by Sinton et al. (1999). The importance of leptin signaling in sleep is indicated by evidence from leptin (ob/ob) and leptin receptor db/db knock-out mice, which display reduced NREM and REM sleep during the rest phase and increased NREM and REM sleep during the active phase, together with sleep fragmentation and reduced delta power at the beginning of the rest phase (Laposky et al., 2006, 2008; Silvani et al., 2009; Pho et al., 2021). These observations are consistent with our results, although the total sleep time was reported to be longer in these animals. Similar observations have been reported for obese human subjects, which are leptin resistant, and display a poor sleep quality during the night and somnolence during the day (Resta et al., 2003; Bixler et al., 2005). Leptin seems to be a sleep promoter, but other factors related to increased adiposity could also contribute, such as the reduction of adiponectin levels. Several prior studies reported a decrease in leptin concentration after sleep restriction in humans (Mullington et al., 2003; Spiegel et al., 2004; Taheri et al., 2004) and rodents (Koban and Swinson, 2005; Martins et al., 2011; Barf et al., 2012); nevertheless, reduced leptin has also been associated with increased time spent in REM sleep (Pan and Kastin, 2014). Leptin and adiponectin carry out their metabolic actions through neurons in the ARC nucleus: leptin activates pro-opio melanocortin (POMC) neurons and inhibits neuropeptide Y (NPY) neurons, in contrast to the effect of adiponectin (Elmquist, 2001; Thundyil et al., 2012; Bouret, 2017), and consistent with our results, NPY increases wakefulness whereas POMC neurons promotes NREM sleep (Szentirmai and Krueger, 2006; Goldstein et al., 2018). Thus, direct and indirect regulation of sleep by adipokines are conceivable.

To explore whether adipokines signaling on the VLPO might affect the activity of other nuclei in the neuronal circuitry involved in sleep and wake regulation, we evaluated c-Fos expression 2 h after vehicle, adiponectin or leptin injection. As expected, adiponectin increased neuronal activity in wake-promoting nuclei, such as TMN and LC. Interestingly, no changes were observed in the contralateral VLPO (Figures 6A,B, 7A,B). Histaminergic TMN cells fire during wake (Jian-Sheng, 2000; Yu et al., 2015) and are silenced by GABA projection from the VLPO during sleep (Sherin et al., 1998). Histaminergic TMN neurons participate in arousal during specific conditions (Venner et al., 2019). The LC is located in the dorsolateral-upper pons and supplies noradrenergic innervation to nearly the entire central nervous system. Ascending projections from noradrenergic LC neurons have been suggested to induce the activation of the whole brain, thus promoting wakefulness (Atzori et al., 2016). The activation of neurons in the TMN and LC after adiponectin injection is consistent with the wake-promoting role reported for this hormone as well as the behavioral data. Adiponectin regulation has been evaluated in a restricted number of nuclei and neurons, such those in the subfornical organ, PVN or POMC and NPY neurons of the ARC, and their response to adiponectin change depending on the energetic state (Hoyda et al., 2007, 2009; Alim et al., 2010; Sun et al., 2016; Suyama et al., 2016). We found that adiponectin increased the activity of OX neurons whereas it reduced the activity of the MCH neurons (Figure 8). These results on neuronal activity are consistent with the increase in wakefulness and the reduction in sleep induced by adiponectin, since OX neurons are essential for wakefulness maintenance whereas MCH neurons promote NREM and REM sleep (Jones and Hassani, 2013; Ono and Yamanaka, 2017).

Leptin administration increased the activity of sleep-promoting neurons in the VLPO whereas it inhibited those in the wake-promoting nucleus TMN (Figure 6). The activation of neurons in the VLPO by leptin is consistent with the increased sleep reported previously (Bjursell et al., 2007). We also detected leptin receptor in the TMN (Supplementary Figure 3), which is consistent with previous reports that found that leptin regulates the activity of histaminergic TMN neurons (Yoshimatsu et al., 1993, 1999; Jochem et al., 2016). Leptin receptors have been reported in the LH-PeF area, specifically in OX and MCH neurons, but also in a different neuronal subset (Wada and Hirako, 2014). We have found that leptin microinjection in the lateral preoptic area modulates LH-PeF neuronal activity, probably by the activation of sleep-active neurons in the VLPO. The decrease of OX activity coupled with the increase in the activity of MCH neurons after leptin microinfusion (Figure 8) is consistent with the reduction in food intake promoted by leptin when administered either centrally or peripherally (Figures 4, 8H–K; Kay et al., 2014; Blasiak et al., 2017). Taken together, these results support the evidence of crosstalk between leptin as an adiposity peripheral signal and the central response of nuclei that facilitate sleep when animals are satiated.

GABAergic mechanisms have been associated with sleep regulation mostly by the hypnotic effects of GABA receptor-mediated drugs (Gottesmann, 2002; Wisden et al., 2019). GAD65 is one of the two isoforms of the protein responsible for GABA biosynthesis and is mainly found in nerve terminals, being considered a GABAergic neuron marker (Dade et al., 2020). The GABAergic and Galaninergic neurons in the VLPO project and inhibit wake-promoting nuclei such as the TMN, LC, and OX neurons in PeF/LH region (Steininger et al., 2001; Chou et al., 2002; Lu et al., 2002; Sakurai et al., 2005; Pierre-Hervé, 2010; Saito et al., 2018). GAD65 immunoreactivity does not change in any area we analyzed except for the increases in LC after adiponectin administration and in the MnPO in the animals which receive leptin microinfusion (Supplementary Figure 7 and Supplementary Tables 1, 2). These results might suggest an increase in the inhibitory input in the LC and MnPO that seems opposite to our hypothesis. These increments could reflect a compensatory mechanism by the interneurons or other GABAergic regions which project to the LC and MnPO in order to reestablish the normal physiological activity at that specific temporal point (Mallick et al., 2001; Jin et al., 2016; Okada and Fukuyama, 2020) or, in the case of the MnPO, regulating different neuronal populations (Vanini et al., 2020; Machado et al., 2022). Nevertheless, the changes in immunostaining are not conclusive and deeper studies evaluating this hypothesis are necessary.

The microinjection of adipokines performed in the preoptic area including the VLPO entails some limitations. The cubical volume that we injected (1 mm^3^) is enough to diffuse and cover completely the VLPO (Bruijnzeel et al., 2013), but is highly possible that the injections reach surrounding preoptic areas expressing adiponectin and leptin receptors, involved in different physiological variables as body temperature (Zhao et al., 2017; Kroeger et al., 2018; Conceição et al., 2019), water consumption (Abbott and Saper, 2017) and Locomotor activity (Subramanian et al., 2018). This might influence the responses we observed in sleep and wake regulation, nevertheless, our conclusions still point to the VLPO as the major responsible due to its relevance to the sleep/wake cycle, in addition to the point that we did not observe significant differences in body temperature potentially attributable to other preoptic regions, and the changes in the neuronal activity on nuclei reported with dense connectivity with the VLPO. Further investigations are necessary to evaluate the specific populations responding to adipokines involved sleep/wake cycle.

Additionally, the strong arousal during the hour of the microinjection due to the handling stress might be a cofounder of the results we obtained during the second hour. Even when most of the variables come back to the basal levels during the second hour after the adipokines administration, the stress might trigger neuronal and humoral mechanism that could influence our results (Li et al., 2020). This could be more critical in the experiments performed during the rest phase of the day, when these mechanisms are reduced, than in the active phase where they reach the high physiological levels. We evaluate the response to leptin and adiponectin according to their lower plasma concentration during the day (so not having the endogenous levels as a cofounder) and the function we hypothesized they could have over the sleep/wake cycle; thus, we cannot directly compare them or evaluate the same response at different temporal points. Finally, due to the number of brain structures analyzed and the immunostaining evaluated, arise a potential inflation of the type 1 error rate that was not corrected in this work. Even though, the adiponectin and leptin effect on different sleep/wake parameters is evident beyond these methodological limitations.

In summary, the present work shows that the main part of the sleep-wake neuronal circuitry responds to hormonal cues secreted by the adipose tissue (adiponectin and leptin) according to the time of the day, in accordance with the energy and sleep requirements. Changes in adiponectin and leptin pattern of secretion that occurs in several metabolic conditions might affect sleep parameters via the VLPO and the other described nuclei. Furthermore, energy and sleep homeostasis might reciprocally affect each other when alterations in feeding behavior and sleep habits occur.

## Author’s note

The present work provides evidence that nuclei that are involved in the control of the sleep-wake cycle are sensitive to metabolic information brought to the brain by the adipokines adiponectin and leptin. We demonstrated that local administration of adiponectin in the VLPO reduces REM sleep and activates wake-promoting neurons in the TMN and LC, while leptin administration increases NREM and REM sleep by inhibition of these brain areas. These results might help explain how abnormal secretion of these adipokines in obesity causes disturbances of sleep and wake physiology.

## Data availability statement

The datasets presented in this study can be found in online repositories. The names of the repository/repositories and accession number(s) can be found below: all data created during this research is openly available in data repository of the Universidad Autónoma de San Luis Potosí at https://repositorioinstitucional.uaslp.mx/xmlui/handle/i/7501.

## Ethics statement

The animal study was reviewed and approved by the Ethical Committee approved all animal care and experimental procedures (CEID2014030), agreeing with the Mexican legislation for animal handling (NOM-062-ZOO-1999).

## Author contributions

RS-D and NS directed the study. OR-P, CP-E, and FG-G performed sleep recording, data collection, and analysis. OR-P, SC-R, and OF-S performed immunohistochemical studies, data collection, and analysis. AB-R, OR-P, and LA-Á performed cannula implantation surgery. OR-P, RS-D, and NS wrote the manuscript. All authors contributed to discussion of the data and edition of the manuscript.

## Conflict of interest

The authors declare that the research was conducted in the absence of any commercial or financial relationships that could be construed as a potential conflict of interest.

## Publisher’s note

All claims expressed in this article are solely those of the authors and do not necessarily represent those of their affiliated organizations, or those of the publisher, the editors and the reviewers. Any product that may be evaluated in this article, or claim that may be made by its manufacturer, is not guaranteed or endorsed by the publisher.

## Supplementary material

Supplementary Figure 1 Positive and negative controls for Adiponectin receptors. **(A)** Western Blots for AdipoR1 and AdipoR2 in brain tissue. Negative controls for AdipoR1 **(B)** and AdipoR2 **(C)** in ARC and PVN, respectively.

Supplementary Figure 2 Immunoreactivity of adiponectin receptors (AdipoR1 and AdipoR2) in the **(A)** SCN, **(B)** MnPO, **(C)** TMN, and **(D)** LC. Dashed lines border the nucleus area.

Supplementary Figure 3 Immunoreactivity of Leptin receptor (LepR) in the **(A)** VLPO and **(B)** TMN. **(C)** Western Blot for LepR in kidney tissue as positive control. Dashed lines border the nucleus area.

Supplementary Figure 4 Daily profile of body temperature before and after **(A)** adiponectin or **(B)** leptin, and vehicle administration. White bar, light phase; dark bar, dark phase; vertical gray line, moment of the adipokine or vehicle administration; **P* < 0.05. Adipokine vs. basal; ^&^*P* < 0.05 vehicle vs. basal.

Supplementary Figure 5 Daily profiles of wake-sleep patterns in adipokines and vehicle administrated groups. **(A,B)** Wake, **(C,D)** NREM, and **(E,F)** REM sleep in adiponectin (left columns) or leptin (right columns) protocols, respectively. White bar, light phase; dark bar, dark phase; dotted line, hour of adipokines administration; **P* < 0.05. Adipokine vs. basal; ^&^*P* < 0.05 vehicle vs. basal; ^#^*P* < 0.05. Adipokine vs. vehicle.

Supplementary Figure 6 Immunoreactive cells to **(A–D)** OX and **(E–H)** MCH after adiponectin or leptin administration.

Supplementary Figure 7 GAD65 immunoreactivity on sleep- and wake-promoting areas. GAD65 immunoreactivity in **(A)** VLPO, **(B)** MnPO, **(C)** TMN, or **(D)** LC after vehicle or adiponectin administration. GAD65 immunoreactivity in **(E)** VLPO, **(F)** MnPO, **(G)** TMN, or **(H)** LC after vehicle and leptin administration.
