# Supplementary figures and images for: Leptin and adiponectin regulate the activity of nuclei involved in sleep-wake cycle in male rats
Source: Front Neurosci. 2022 Jul 22;16:907508. doi: 10.3389/fnins.2022.907508 (PMC9355486; doi:10.3389/fnins.2022.907508)

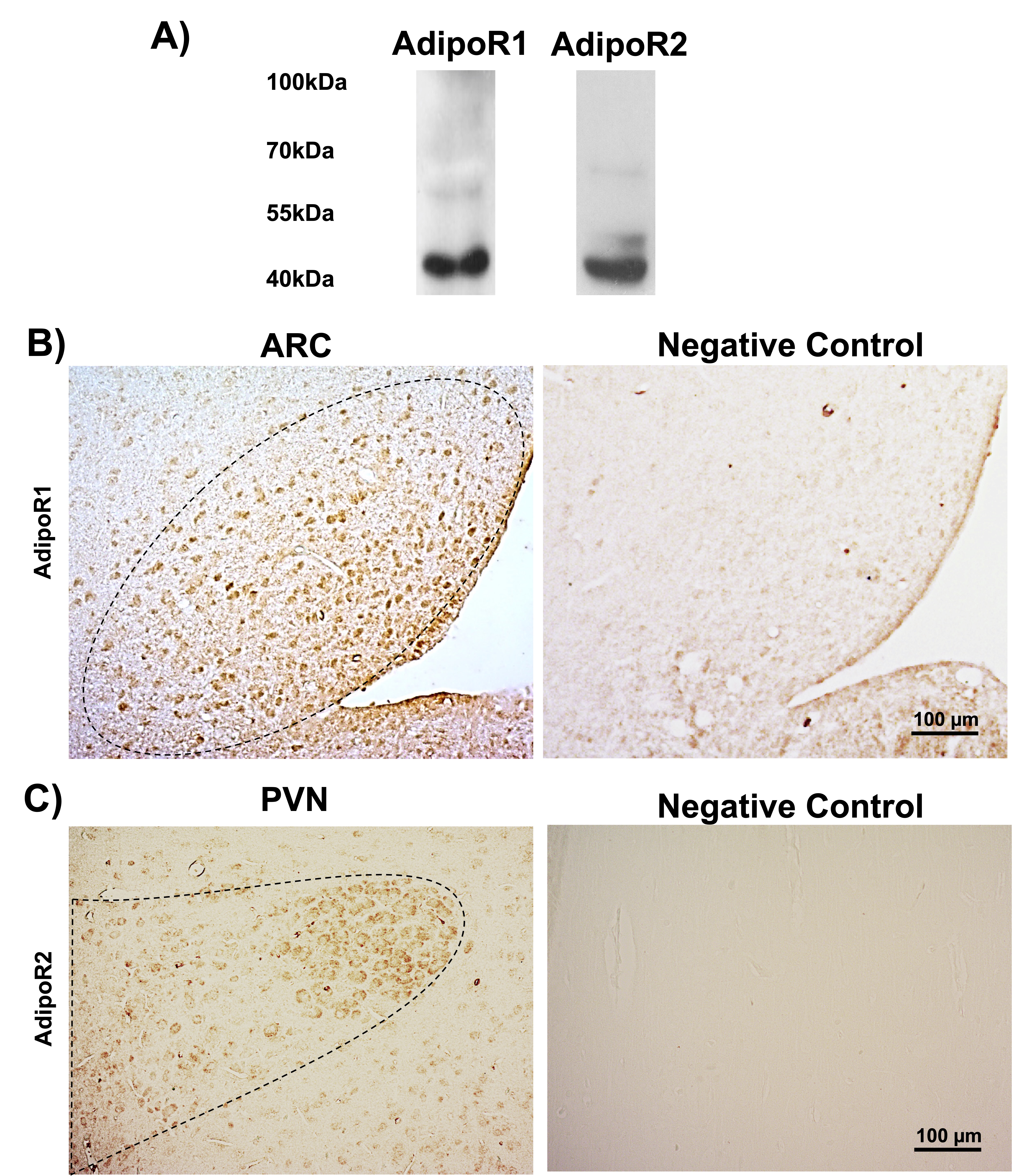

Supplement: Supplementary Figure 1 — Positive and negative controls for Adiponectin receptors. (A) Western Blots for AdipoR1 and AdipoR2 in brain tissue. Negative controls for AdipoR1 (B) and AdipoR2 (C) in ARC and PVN, respectively. [file Image_1.JPEG]

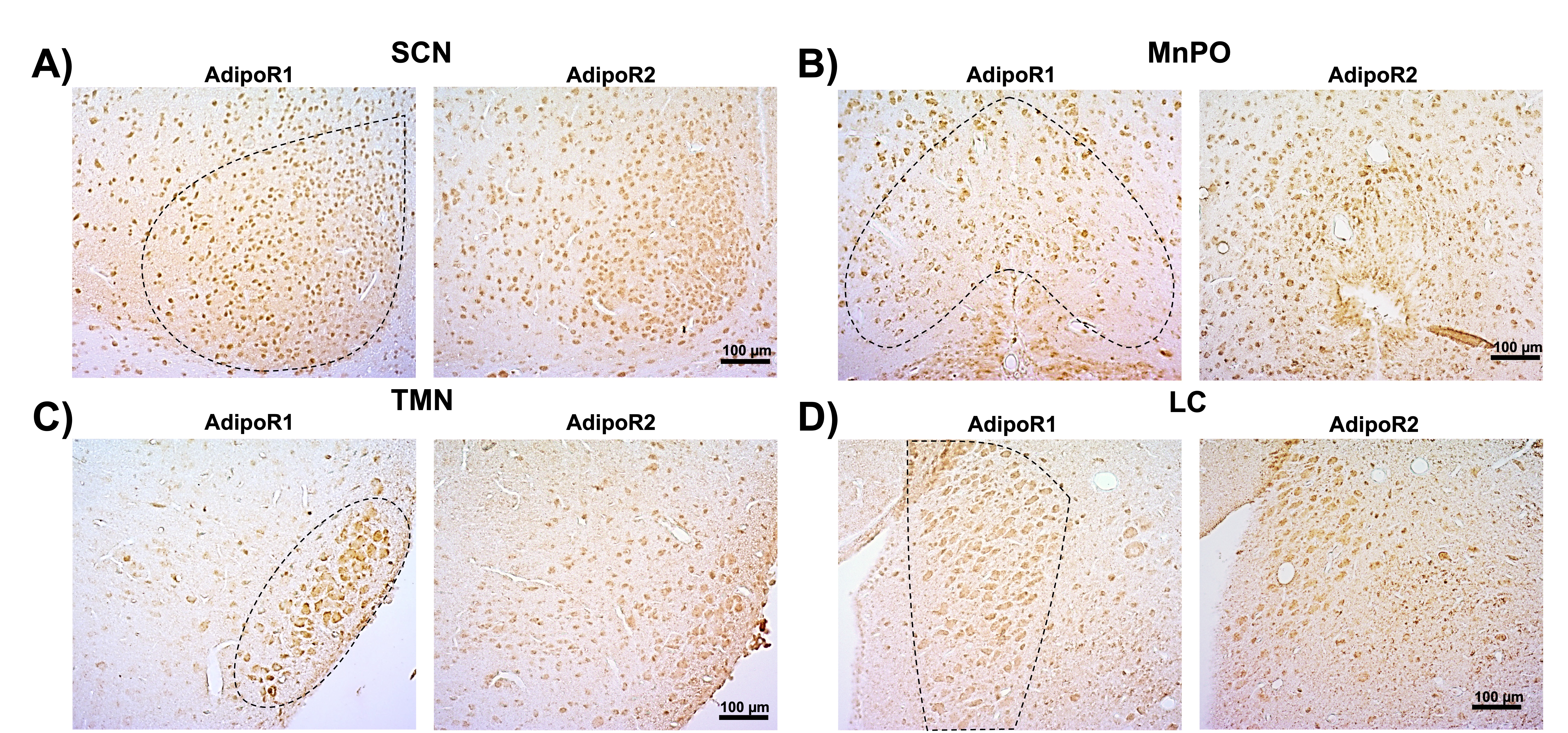

Supplement: Supplementary Figure 2 — Immunoreactivity of adiponectin receptors (AdipoR1 and AdipoR2) in the (A) SCN, (B) MnPO, (C) TMN, and (D) LC. Dashed lines border the nucleus area. [file Image_2.JPEG]

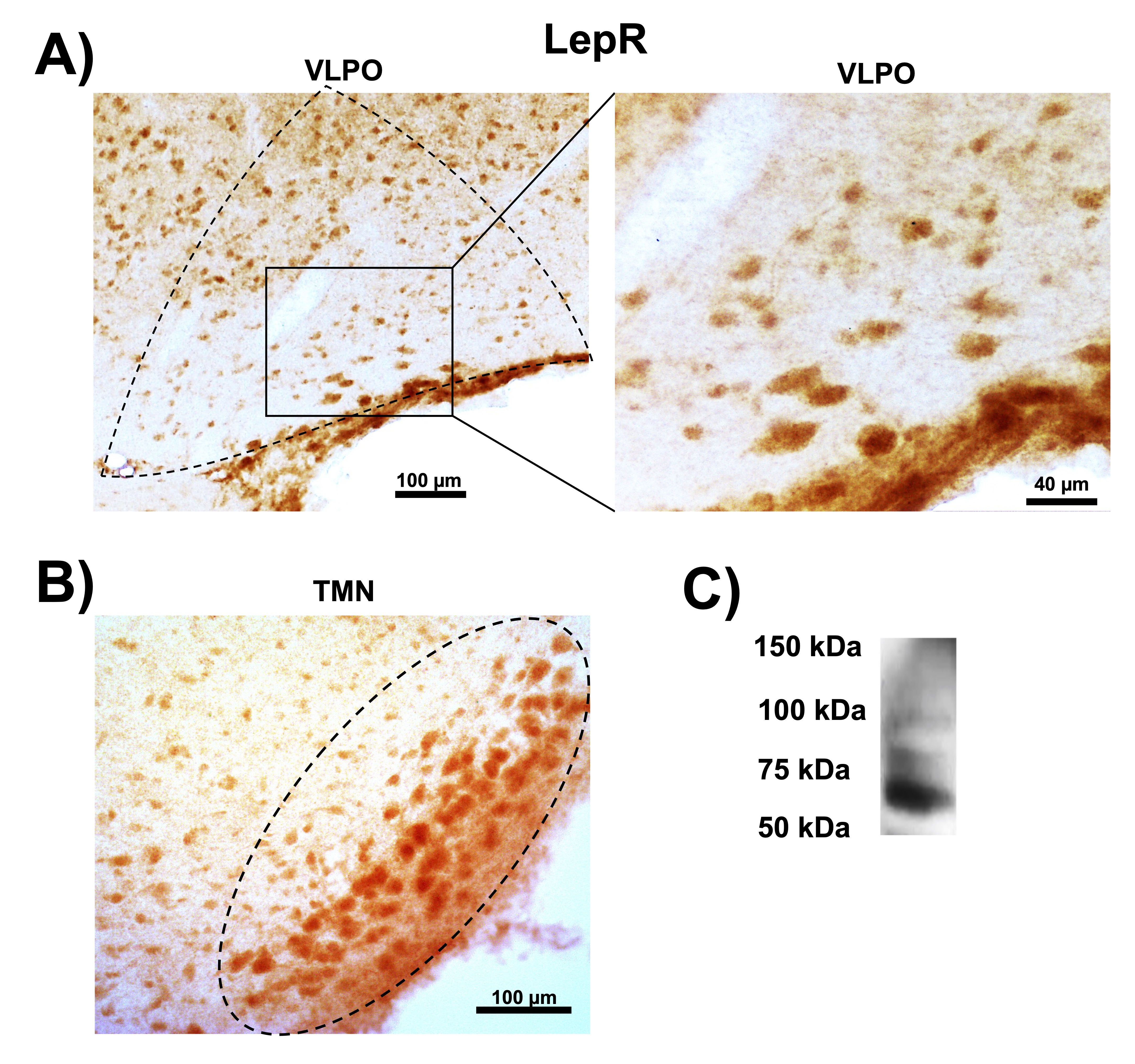

Supplement: Supplementary Figure 3 — Immunoreactivity of Leptin receptor (LepR) in the (A) VLPO and (B) TMN. (C) Western Blot for LepR in kidney tissue as positive control. Dashed lines border the nucleus area. [file Image_3.JPEG]

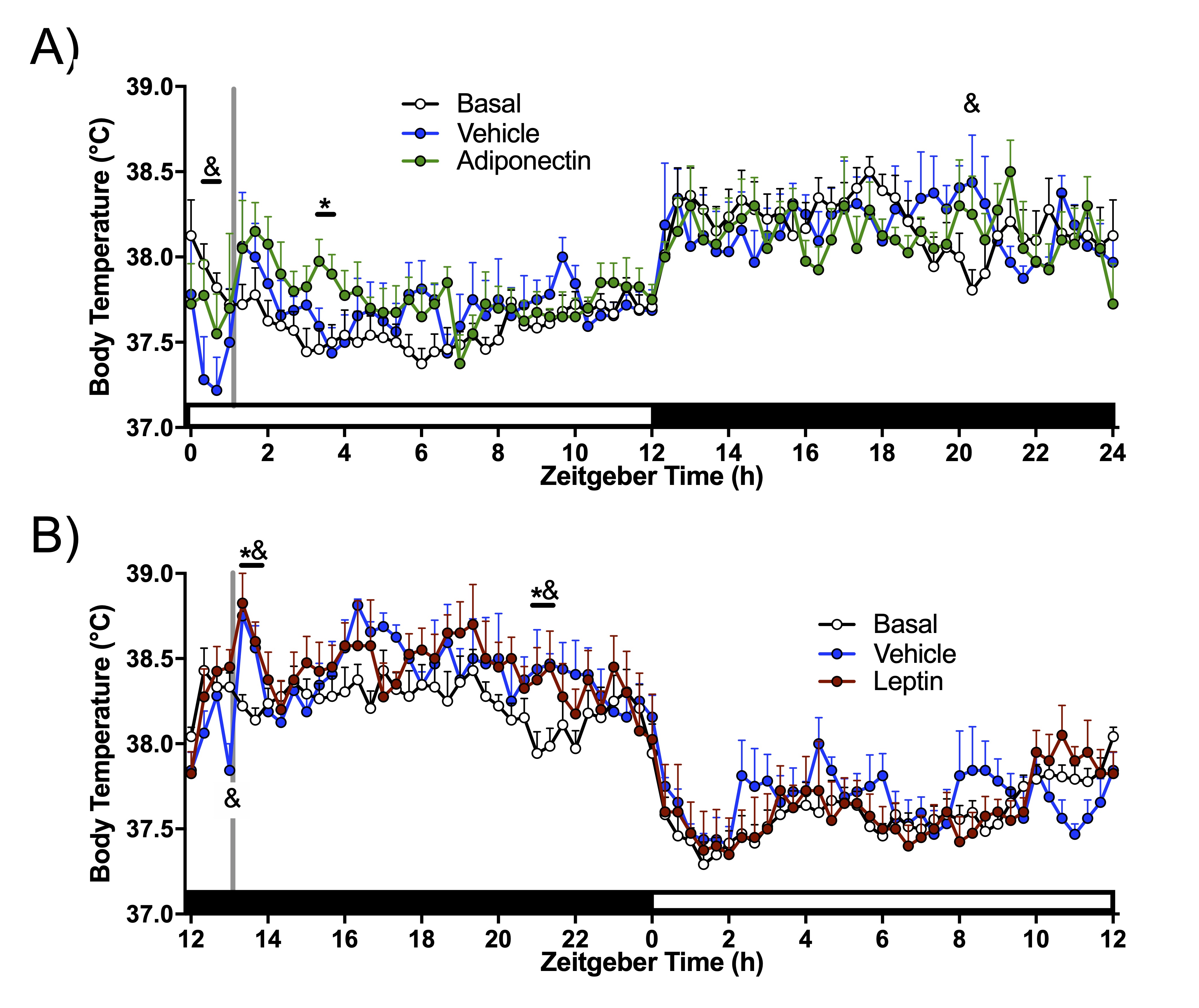

Supplement: Supplementary Figure 4 — Daily profile of body temperature before and after (A) adiponectin or (B) leptin, and vehicle administration. White bar, light phase; dark bar, dark phase; vertical gray line, moment of the adipokine or vehicle administration; *P < 0.05. Adipokine vs. basal; &P < 0.05 vehicle vs. basal. [file Image_4.JPEG]

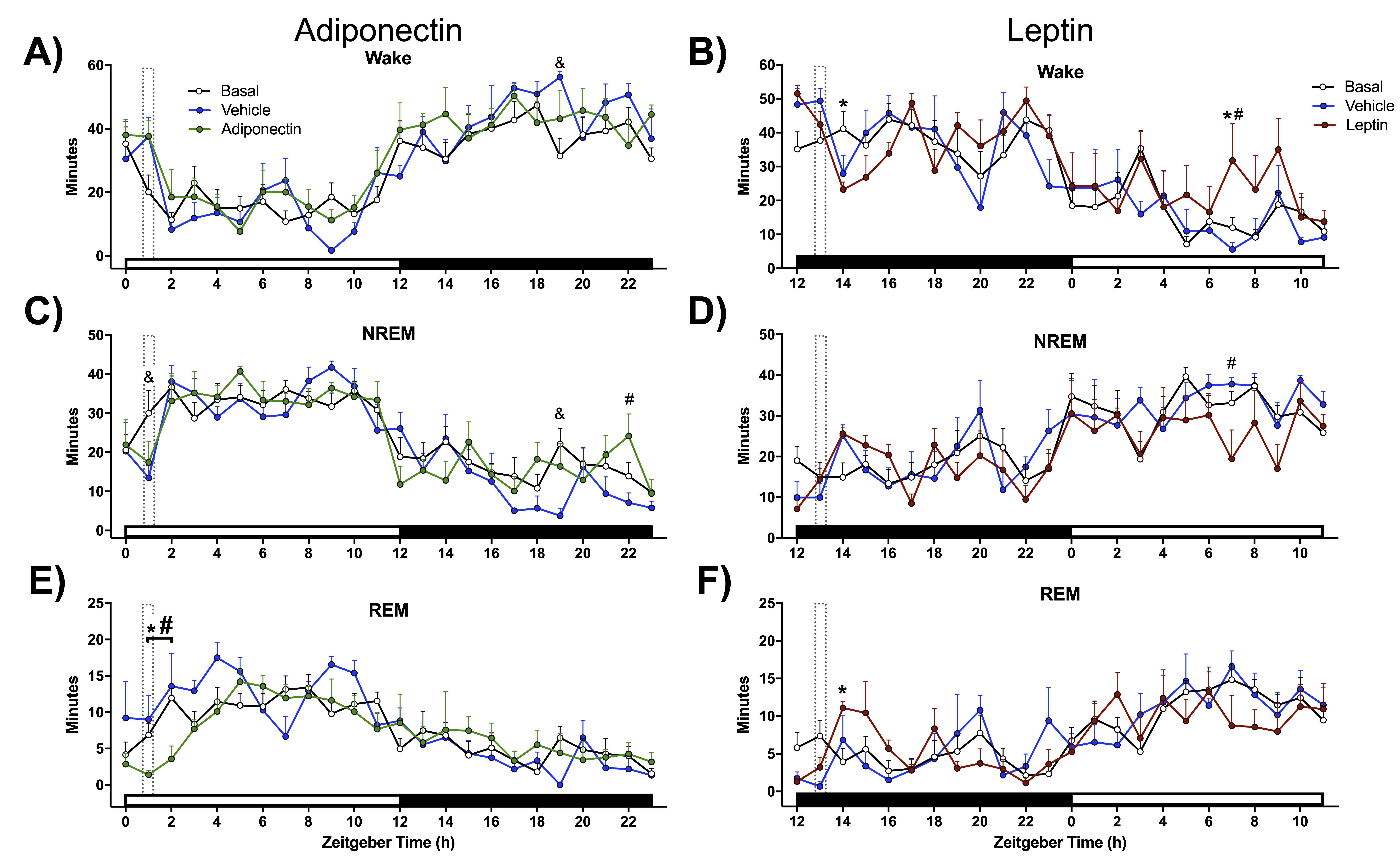

Supplement: Supplementary Figure 5 — Daily profiles of wake-sleep patterns in adipokines and vehicle administrated groups. (A,B) Wake, (C,D) NREM, and (E,F) REM sleep in adiponectin (left columns) or leptin (right columns) protocols, respectively. White bar, light phase; dark bar, dark phase; dotted line, hour of adipokines administration; *P < 0.05. Adipokine vs. basal; &P < 0.05 vehicle vs. basal; #P < 0.05. Adipokine vs. vehicle. [file Image_5.JPEG]

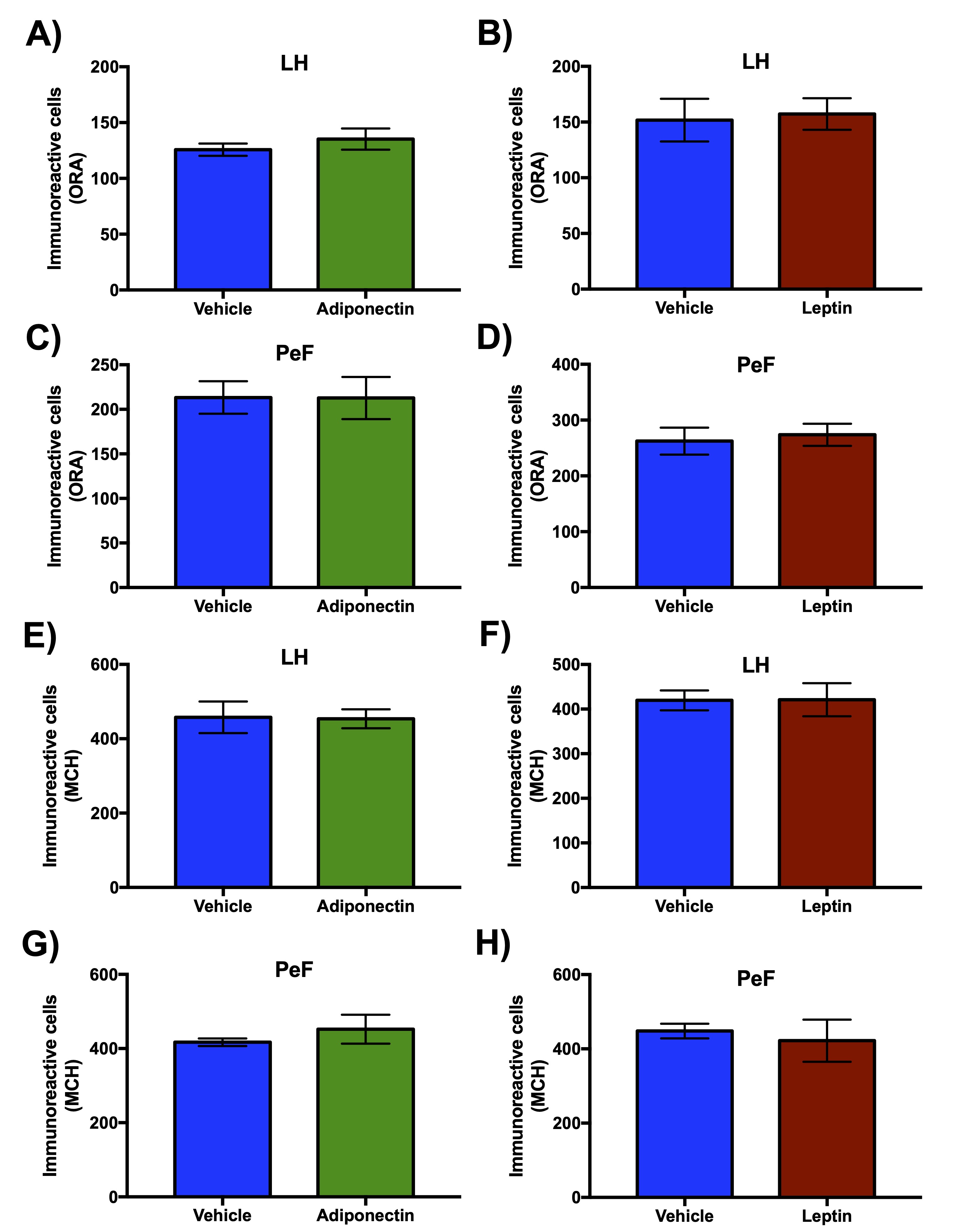

Supplement: Supplementary Figure 6 — Immunoreactive cells to (A–D) OX and (E–H) MCH after adiponectin or leptin administration. [file Image_6.JPEG]

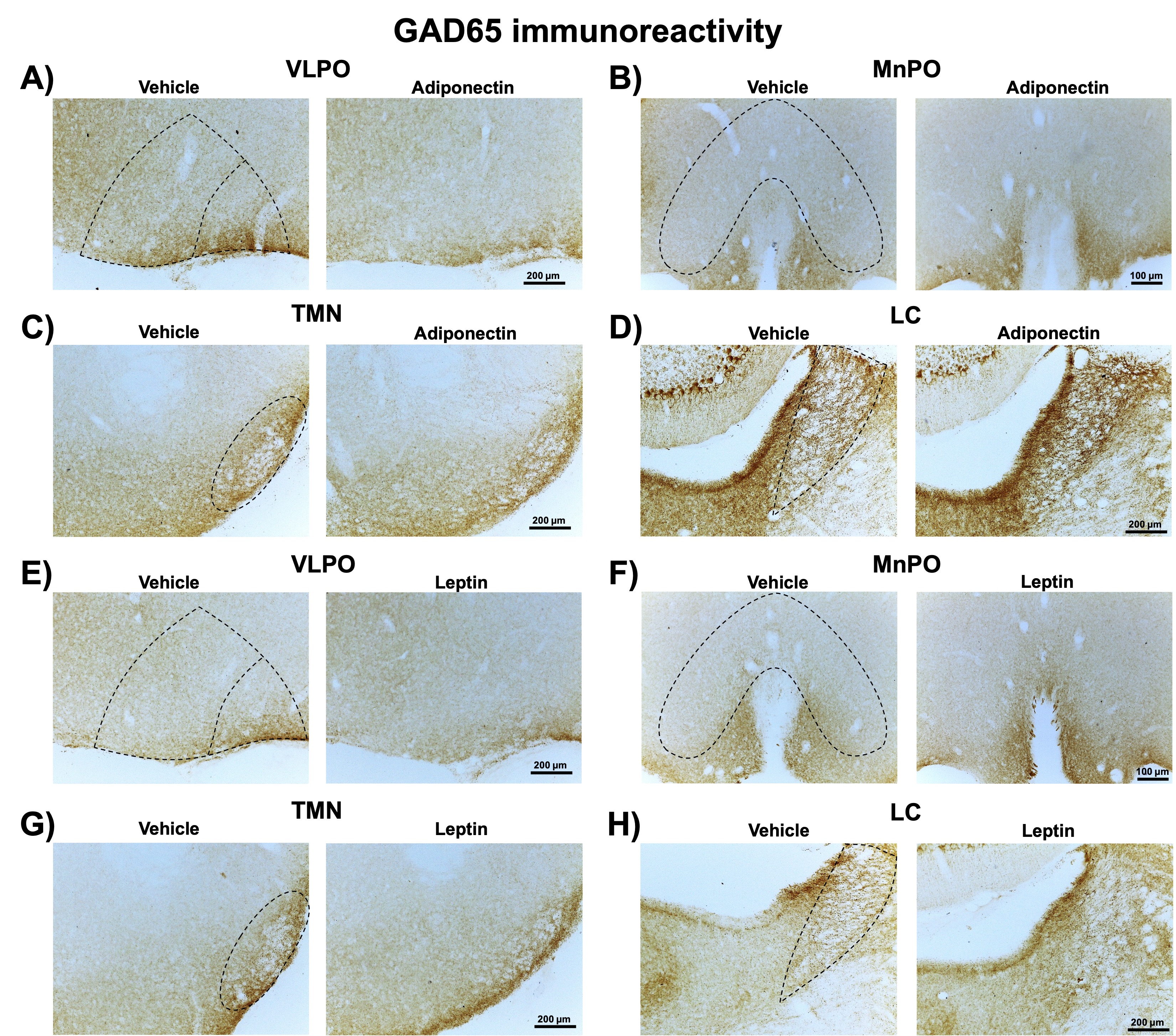

Supplement: Supplementary Figure 7 — GAD65 immunoreactivity on sleep- and wake-promoting areas. GAD65 immunoreactivity in (A) VLPO, (B) MnPO, (C) TMN, or (D) LC after vehicle or adiponectin administration. GAD65 immunoreactivity in (E) VLPO, (F) MnPO, (G) TMN, or (H) LC after vehicle and leptin administration. [file Image_7.JPEG]
